# Anatomical orientation lines for localization of the transseptal puncture site in a 3D electroanatomical map

**DOI:** 10.1007/s10840-023-01571-3

**Published:** 2023-05-30

**Authors:** Khuraman Isgandarova, Martin Braun, Vanessa Sciacca, Thomas Fink, Mustapha El Hamriti, Moneeb Khalaph, Denise Guckel, Christian Sohns, Philipp Sommer, Guram Imnadze

**Affiliations:** Clinic for Electrophysiology, Herz- und Diabeteszentrum NRW, Ruhr-Universität Bochum, Georgstr. 11, 32545 Bad Oeynhausen, Germany

## Introduction

Transseptal puncture (TSP) is the main access route during left-sided ablations. Fluoroscopy is still the most common guidance tool [1]. However, for localization of the fossa ovalis (FO), transesophageal or intracardiac echocardiography can be used [2, 3]. Considering the fact that little is known about the topic, we evaluated the usefulness of anatomical landmarks reconstructed on the 3D map of the right atrium for detecting the posterior border for the optimal TSP site.

## Methods

### Patients and preprocedural management

One hundred patients who underwent an ablation for paroxysmal or persistent atrial fibrillation in our center between June 2020 and November 2021 were prospectively enrolled.

The ablation procedure was performed under deep analgosedation. A 6F decapolar catheter Inquiry™ (Abbott, St. Paul, MN, USA) was inserted into the right atrium (RA), and an accurate three-dimensional electroanatomical map (EAM) using the EnSite Precision system (Abbott, St. Paul, MN, USA) was obtained. TSP was performed under fluoroscopic control. For more precise anatomical orientation, the reconstructed 3D multislice-detector computed tomography images were integrated into the EAM.

### Postprocedural analysis

As published before, we have marked the CS (coronary sinus)–SVC (superior vena cava) line from the anterior margin of the CS ostium (mid-point) to the anterior margin of the SVC directly at the highest transition point to the right atrial appendage in LAO projection [4]. The difference is that Eichenlaub et al. used the magnetic field–based 3D mapping system for this purpose. In contrast, in our study, the electrical impedance field–based 3D mapping system was used. After marking the anterior line, RA geometry was then angulated towards LAO/LLat until the CS–SVC line was strictly vertical, and then the map was cranialized to a 30° angle, into the plane view of the FO. In this angulation, we marked two additional lines as follows:

- SVC line: from an anterior margin of SVC at the transition into RA/RAA to the posterior margin of IVC at the transition into RA
- IVC (inferior vena cava) line: from an anterior margin of IVC at the transition into RA to the posterior margin of SVC at the transition to RA

**Fig. 1:**
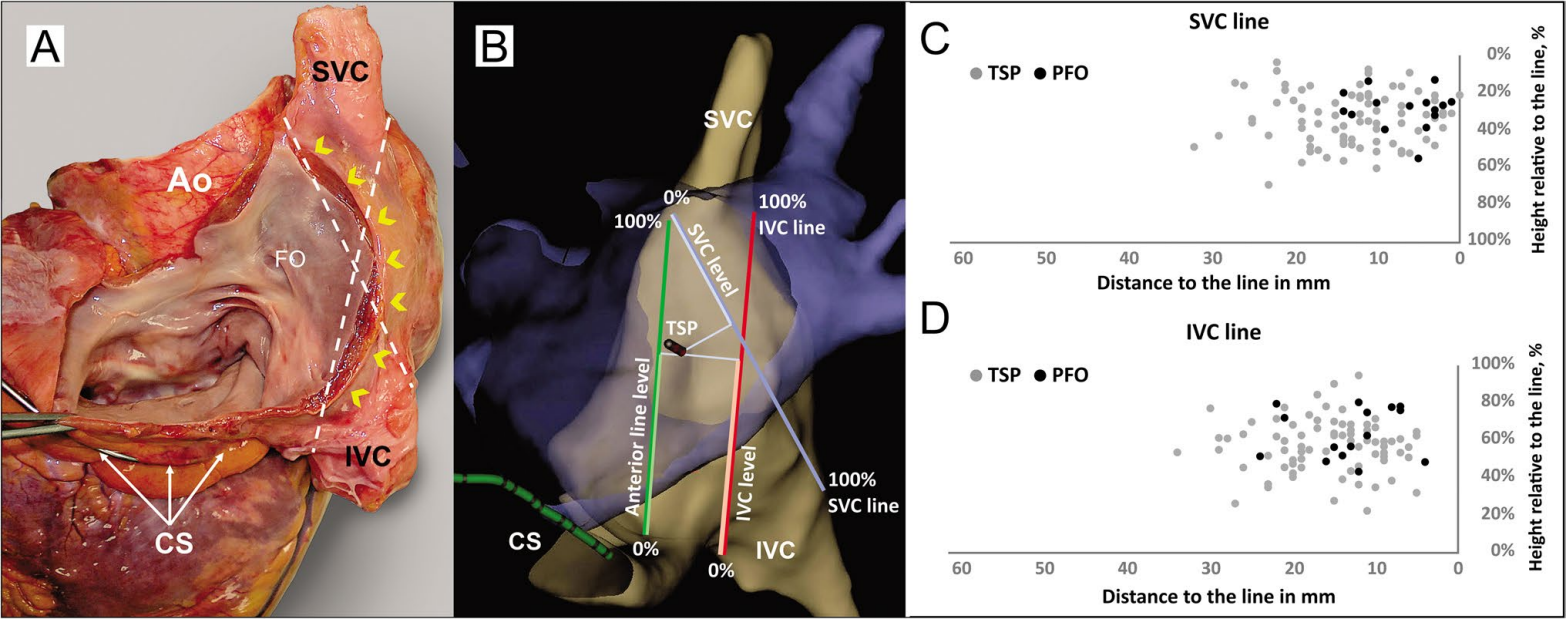
**A** Anatomical specimen showing the structures of interatrial septum from the left atrial side. Dotted lines describes the SVC and IVC lines; the yellow arrowheads along the Waterstones groove. **B** 3D-electroanatomical map of the right atrium (gray) and the integrated CT scan of the left atrium in blue. Schematical illustration of additional anatomical measurements: Anterior; SVC and IVC lines; distances from the TSP to the lines as well as level of the TSP according to the line (measured in percentage): anterior line level; SVC level and IVC level. **C** and **D** Scatter plots demonstrating TSP sites in relation to the lines. SVC, superior vena cava; IVC, inferior vena cava; TSP, transseptal puncture (gray dots); PFO, persistent foramen ovale (black dots). FO, fossa ovalis; Ao, ascending aorta; IVC, inferior vena cava; SVC, superior vena cava; CS, coronary sinus; TSP, transseptal puncture

We measured the distance and height of the TSP or persistent foramen ovale (PFO) relative to the abovementioned lines and also to the CS ostium (the most superior part). The height was measured as a percentage relative to the length of the lines. Distances were measured relative to their perpendicular point of intersection with the lines (Fig. 1).

### Statistical analysis

A Student’s test was used for the comparison of continuous variables. A two-tailed *p*-value < 0.05 was considered statistically significant. All calculations were performed using the IBM SPSS Statistics 27.0, NY, USA.

## Results

We have included 100 consecutive patients who underwent ablation due to paroxysmal (*n* = 34, 34%) or persistent atrial fibrillation (*n* = 66, 66%). The average age of patients was 66 ± 11 years, 73 patients (73%) were male. Further baseline characteristics are listed in Table 1.

**Table 1:** Baseline characteristics and postprocedural analysis

	Measurement
Baseline characteristics	
Age, years	66 ± 11
Sex (males)	73 (73%)
Arterial hypertension	65 (65%)
BMI, kg/m^2^	28 ± 6
BSA, m^2^	2 ± 0.3
DM	13 (13%)
SHD	33 (33%)
LVEF, %	55 ± 4
LA-diameter in CT, mm	48 ± 11
AF type	
Paroxysmal	34 (34%)
Persistent	66 (66%)
AF duration, years	2 ± 0.5
Re-do procedure	34 (34%)
Postprocedural analysis	
Anterior line	
Length anterior line, mm	50 ± 11
Distance from TSP site to the anterior line, mm	8 ± 5
Height of TSP site relative to anterior line, %	55 ± 1
SVC line	
Length SVC line, mm	53 ± 11
Distance from TSP site to SVC line, mm	12 ± 7
Height of TSP site relative to SVC line, %	31 ± 1
IVC line	
Length IVC line, mm	58 ± 12
Distance to IVC line, mm	15 ± 6
Height of TSP site relative to IVC-line, %	56 ± 1
CS distance, mm	15 ± 8

*BMI* body mass index, *BSA* body surface area, *DM* diabetes mellitus, *LVEF* left ventricular ejection fraction, *SHD* structural heart disease, *LA* left atrium, *AF* atrial fibrillation, *SVC* superior vena cava, *TSP* transseptal puncture, *IVC* inferior vena cava, *CS* coronary sinus. Values are given as a median ± standard deviation

All TSP sites were posterior to the anterior line (mean distance—8 ± 5 mm). The TSP site was found to be predominantly on the superior level of the SVC line and was at the mean distance of 12 ± 7 mm of it (Table 1). The TSP site in one patient was on the SVC line. All TSP sites were separated from the IVC line at the mean distance of 15 ± 6 mm and tended to be projected on the middle level of the IVC line. All sites of the transseptal passage were above the CS level, and the mean distance to it was 15 ± 8 mm (Fig. 1).

## Discussion

The main findings of the current study are:

1. The electrical impedance field–based 3D mapping system appears to have the same potential benefits for localization of the anterior border of the transseptal puncture site as the magnetic field–based 3D mapping system.
2. The proposed anatomical lines provide accurate localization of the posterior border of the TSP area.

In recent publications, colleagues used the magnetic field–based 3D mapping system and proved that the anterior line demarcates the anterior border of FO and can be used for guiding the TSP [4, 5]. However, they did not provide information about landmarks which demarcates the posterior border of the FO. Once TSP is produced too posteriorly, towards the Waterstone groove, a major complication can occur.

In our study, we were able to show that the anterior borderline of FO described before is reproducible also when using a 3D navigation system based on electrical impedance. Moreover, the new additional lines of SVC and IVC corresponded to the posterior boundaries of the FO region. The intersecting character of the SVC and IVC lines reproduces the convex nature of the Waterstone groove. No TSP site was projected on the outer side of the presented lines; in other words, TSP was always inside of the geometrical figure built by the aforementioned lines. The clinical significance of these lines for TSP and their effect on reducing fluoroscopy time will be explored in ongoing studies.

New technical achievements, like visualized sheaths and RF needle as well as the utilization of three-dimensional intracardiac echocardiography, are getting more attention in terms of fluoroscopy time reduction [6–8]. Therefore, geometric localization of the FO using the aforementioned lines could be another step towards minimizing the fluoroscopy. All described methods need to be further evaluated and compared in large, randomized trials. However, fluoroscopy remains the main method of TSP so far.

## Study limitation

The presented data was collected from a single center with a relatively small number of patients.

## Conclusion

The novel SVC and IVC lines together with the anterior line based on a 3D mapping system are corresponding to the TSP site. Further study is needed to prove safety and/or influence on the fluoroscopy time reduction once using these landmarks.

## Data Availability

Data will be made available from the authors on reasonable request.
